# Arthroplasty and Tennis: A Narrative Review

**DOI:** 10.1177/23259671251397401

**Published:** 2025-12-08

**Authors:** Peter Kaiser, Johannes Neugebauer, Felix Riechelmann, Friedemann Schneider, Todd S. Ellenbecker, Alexander Keiler

**Affiliations:** * University Hospital for Orthopaedics and Traumatology, Medical University of Innsbruck, Innsbruck, Austria; † Sportklinik Arlberg GmbH, St. Anton am Arlberg, Austria; ‡ Division of Orthopaedics and Traumatology, University Hospital Krems, Karl Landsteiner University of Health Sciences, Dr. Karl-Dorrek-Straße 30, 3500, Krems, Austria; ‖ Banner Sports Medicine & ATP Tour, Scottsdale, Arizona, USA; Investigation performed at University Hospital for Orthopaedics and Traumatology, Medical University of Innsbruck, Innsbruck, Austria

**Keywords:** sports, tennis, joint replacement, arthroplasty, physical therapy/rehabilitation

## Abstract

**Background:**

An active sporting lifestyle is associated with numerous health benefits. In this regard, tennis as a sport that can be practiced by young and old can be a component throughout life. The extent to which play is possible and medically advisable after arthroplasty of the hip, knee, ankle, and shoulder joint is the subject of this narrative review.

**Purpose:**

To summarize the current literature and evidence regarding prosthetic treatment of the hip, knee, ankle, and shoulder joints in relation to tennis, both in singles and in doubles, so the clinician can use evidence-based medicine to advise and treat patients who want to return to tennis.

**Study Design:**

Narrative review.

**Methods:**

An electronic databases search, including PubMed, Google Scholar, ScienceDirect, UpToDate, and Springer, was conducted on articles including tennis and arthroplasty.

**Results:**

The return-to-play rate ranged from 7% to 62.5% for total hip arthroplasty, 0% to 62.5% for total knee arthroplasty, 0% to 37.5% for unicondylar knee arthroplasty, 0% to 30% for total ankle arthroplasty, 75% to 100% for anatomic total shoulder arthroplasty, 0% to 50% for reverse total shoulder arthroplasty, and 50% to 100% for shoulder hemiarthroplasty. Tennis play clearance given by surgeons (for singles/doubles) was 80%/89% for total hip, 70%/87% for total knee, 40%/85% for total ankle, 75%/86% for anatomic total shoulder, 30%/43% for reverse total shoulder, and 85/88% for shoulder hemiarthroplasty. The mean period for a return to play was 5-7 months (range, 1-36 months). Existing evidence cannot prove a higher complication rate caused by playing tennis.

**Conclusion:**

The current evidence indicated that tennis can be continued even after arthroplasty. The return-to-play rate was higher for total hip arthroplasty, total knee arthroplasty, anatomic shoulder arthroplasty, and shoulder hemiarthroplasty compared with ankle and reverse shoulder arthroplasty. The time frame for a return to sports depended on a variety of intrinsic and extrinsic factors. For players with sufficient experience, tennis doubles and singles could be played after 3 to 6 months. Each patient should therefore be assessed and advised individually and also informed that the practice of high-impact sports (such as tennis) could potentially lead to complications like premature polyethylene wear, implant loosening, dislocations, or periprosthetic fractures, even though no fully conclusive data are available. This article provides information about the outcomes and return-to-play rates for tennis after shoulder, hip, knee, and ankle replacement and will help physicians better advise patients who wish to return to tennis after endoprosthesis surgery.

According to the International Tennis Federation *Global Tennis Report of 2019*,^43^ approximately 1.17% of the world's population (87 million) participate in tennis. The number of members of the German Tennis Association has risen from just under 71,000 in 1948 to just under 1.5 million (2023), with significantly more people playing tennis than there are listed members.^22^

Whereas swimming, cycling, golf, and walking are referred to as low-impact sports, tennis is typically classified as a high-impact sport.^84^ An in vivo study showed that the mean peak force exerted on a total knee arthroplasty (TKA) was about 3.6 times body weight for the serve and forehand and 3.1 times body weight for the backhand.^24^ The anteriorly directed shear force, however, was quite low at 0.28 times body weight.^24^ Despite the high force, the mode of play must be taken into account in addition to the individual style of play. Often, a distinction is made between singles and doubles play. Although the strain in singles with increased amounts of running and changes of direction can be very high, the strain in doubles with more volleys and shorter amounts of running is significantly lower. Therefore, many authors classify singles tennis as a high-impact sport and doubles tennis as a low-impact sport, which makes a homogeneous assessment difficult. It is also postulated that the individual style of play is decisive for the actual assessment.^38^ In terms of the upper extremity, however, both singles and doubles tennis can be seen as high-impact sports (which primarily affect the dominant shoulder).^28,31^

Tennis is played with equal passion by young and old. However, older players report pain more frequently.^58^ If such pain is caused by degenerative joint diseases (arthrosis), joint replacement is increasingly used.^41,71^

Although relieving pain and maintaining mobility in daily life were previously the main focuses of therapy, the therapeutic focus has been placed increasingly on maintaining a high postoperative level of sport, especially for younger patients receiving prosthetic treatment.^3^ This change in focus raises questions as to what extent and how quickly tennis can be played again after a prosthetic joint replacement. This review aims to summarize the current literature and evidence regarding prosthetic treatment of the hip, knee, ankle, and shoulder joints in relation to tennis, both in singles and in doubles, so the clinician can use evidence-based medicine to advise and treat patients who want to return to tennis.

## Methods

A literature search including PubMed, Google Scholar, ScienceDirect, UpToDate, and Springer was conducted on articles including tennis and arthroplasty. The search terms “tennis” and/or “hip,”“knee,”“shoulder,”“ankle,” and “replacement” or “arthroplasty” were used. After the abstract was checked, applicable studies that reported on tennis and arthroplasty were read, and data were included in this narrative review. Additional references and papers were extracted from these articles if applicable and not found previously. Papers that were not accessible for full text reading after checking the abstract were dismissed.

### Return to Play

The return-to-play (RTP) rate differed significantly between the locations of the artificial joints mentioned. The range of data was very broad, and the number of cases in the individual studies was often very low. Therefore, the quality of the data can be assessed only to a limited extent.

A study that reported on patients after TKA, total hip arthroplasty (THA), hip resurfacing, unicondylar knee arthroplasty (UKA), and patellar resurfacing in a pooled form showed that 15 of 40 patients (37.5%) were unable to return to tennis and 62.5% played tennis again postoperatively.^97^ The study found no connection between the surgical methods in the pooled calculation among all the sports examined. However, it is hard to interpret the data in a meaningful way given that the age, activity levels, and specific pathologies given widely varied.^97^

Regarding the shoulder joint, a distinction is made between a shoulder hemiarthroplasty (SHA), an anatomic total shoulder arthroplasty (a-TSA), and a reverse total shoulder arthroplasty (r-TSA). The published data indicated that a return to tennis was more likely after THA, TKA, a-TSA, and SHA than after a total ankle arthroplasty (TAA) and r-TSA. In some cases, the percentage probability of returning to tennis was very low (Table 1). Patients should therefore be given detailed information to set realistic expectations. This applies in particular to TAA and r-TSA, which often make a return to tennis impossible.^28,44^ A differentiation between singles and doubles play as well as the hitting arm versus the nondominant side was not possible with regard to the percentages in Table 1 due to the low level of evidence and poor data. Neither did any study address the performance of a 1-handed or 2-handed backhand. These are very important topics because they highly influence the return rate and playing performance. Without such data it is hard to present meaningful conclusions. Although tennis must be considered high impact for the hitting arm in the area of shoulder prostheses, this does not apply to the nondominant side, since in the case of a 1-handed backhand there is hardly any relevant force acting on the shoulder.

**Table 1:** Return-to-Play Rates After Arthroplasty

Arthroplasty Type	Range, %	% of Patients (n/N)*^*a*^*
Total hip arthroplasty	7-62.5	7% (1/14)^14^ 20% (3/15)^70,79^ 21% (22/103)^75^ 22%^*b*^ (10/46)^40^ 36% (5/14)^42^ 44% (4/9)^26^ 62.5%*^*c*^* (25/40)^97^
Total knee arthroplasty	0-62.5	0% (0/2, 0/10)^7,92^ 14% (2/14)^15^ 18% (2/11)^95^ 20% (6/30, 11/56)^11,76^ 33%*^*d*^* (2/6)^40^ 43% (27/63)^51^ 62.5%*^*c*^* (25/40)^97^
Unicondylar knee arthroplasty	0-37.5	0% (0/5, 0/3)^94,77^ 14% (3/21)^65^ 17% (6/31)^73^ 37.5% (3/8)^95^
Total ankle arthroplasty	0-33	Singles 33% (2/6); doubles 0% (0/5)^66^ 18% (7/38)^10^ 29% (2/7)^81^
Anatomic total shoulder arthroplasty	75-100	100% (3/3 1× hitting arm/2× nondominant side)^82^ Singles 87% (80% hitting arm/100% nondominant side) (7/8); doubles 80% (75% hitting arm/100% nondominant side) (4/5)*^*e*^*^30^ Singles 80% (4/5); doubles 100% (4/4)^29^ 100%^67^
Reverse total shoulder arthroplasty	0-50	0% (0/2)^28^ 50% (15/30)^21^ Singles 25% (3/12); doubles 25% (2/8)^32^ 38% (8/21)^48^ Singles 33% (4/12); doubles 38% (3/8)^52^
Shoulder hemiarthroplasty	50-100	Singles and doubles 100% (3/3 each)^29^ Singles 50% (4/8); doubles 57% (4/7)*^*f*^*^31^ Singles 60% (3/5); doubles 67% (4/6)^52^
Shoulder arthroplasty*^*g*^*	63.5-75	63.5% (35/54)^1^ 66% (47/75)^53^ 69% (34/58)^72^ 75% (21/28)^59^

a Values are for singles unless otherwise indicated.

b Compared with lifetime activity; 300% compared with preoperative activity.

c Pooled data after total hip arthroplasty, total knee arthroplasty, hip resurfacing, unicondylar knee arthroplasty, and patellar resurfacing.

d Compared with lifetime activity; 200% compared with preoperative activity.

e Data for players <55 years.

f Higher rate after shoulder hemiarthroplasty in tennis players <66 years: 67% singles, 80% doubles.

g Pooled data with different shoulder prosthesis types.

The RTP rate differed significantly depending on the type of prosthesis. For example, although 0 of 2 players (0%) were able to play tennis again after r-TSA,^28^ the number was significantly higher after SHA, where return to tennis was possible for 3 of 3 players (100%). However, we must consider the low patient count in this study.^29^ Other authors observed an equal RTP rate for SHA and r-TSA, for singles and doubles tennis. However, this was lower than reported results for a-TSA.^52^ The RTP rate after revision surgery following r-TSA was very low (singles 25%/doubles 33%).^32^

Playing tennis was still possible after TSA^12^ or r-TSA.^13,85^ The RTP for tennis appeared to be somewhat higher for players younger than 66 years (SHA) or younger than 70 years (r-TSA) than for older players.^31,32^ Only about 24% were able to play tennis during the time they suffered from their shoulder pathology before surgery.^48^ However, it cannot be ruled out that singles and doubles tennis (n = 1 each) may begin only after r-TSA.^32^

Regarding TKA, 77% of players were either satisfied or very satisfied with their return to tennis, whereas 18% were unsatisfied or very unsatisfied and 8% were indifferent.^51^ One reason may be a decline in their playing performance, which was reported by 39%; 33% reported an increase in their playing performance after TKA in comparison to before surgery.^51^

The most common reason for patients not returning to their respective sport was the recommendation of the treating surgeon to refrain from the specific sport.^86^ Other reasons given by patients included pain, the inability to perform sport-specific movements, fear of damaging the joint, a lack of self-confidence, a lack of interest in playing, or problems with the prosthesis.^32,97^ Medical problems independent of the artificial joint can also prevent RTP.^28^ However, the likelihood of returning to the sport one played before surgery appeared to depend less on age than on sex: Men were almost twice more likely to RTP than women.^97^

The high kinetic load on the arm during the serve has been postulated to be the reason for a low RTP after a shoulder replacement. However, one could also play tennis without serving, so the ability to perform a forehand and backhand is probably also limited.^21,91^ This has so far not been investigated in the existing literature.

Due to the divergent and sometimes low RTP rates, questions arise as to the recommendations of surgeons or professional societies. Playing tennis after THA and TKA was prohibited by surgeons and study statements in the 1980s and early 1990s, but this has changed significantly.^84^ Many societies and surgeons now differentiate between singles and doubles and take other individual characteristics into account, such as playing ability, experience, or body mass index.^35,54,87,88^

Furthermore, timing of the return to tennis has been examined more closely in different studies.^87,88^ Thaler et al^88^ evaluated questionnaires from the European hip and knee societies and showed that a large proportion of respondents allowed both singles and doubles tennis after HA (Table 2). Surgeons who used a larger head (≥36 mm) for THA were more likely to allow singles tennis than surgeons who used a smaller head.

**Table 2:** Surgical Recommendation to Play Tennis After Total Hip Replacement*^*a*^*

Activity and Postoperative Time Point	Yes*^*b*^*	No*^*c*^*	Allowed	Allowed With Experience	Not Allowed	No Opinion	Recommendation
Singles tennis							
6 wk	17	**84**	6	11	81	3	Not recommended
6-12 wk	48	**51**	23	25	47	4	Not recommended
12 wk to 6 mo	**72**	28	49	23	21	7	Undecided
>6 mo	**80**	20	65	15	13	7	Recommended
Doubles tennis							
6 wk	23	**77**	10	13	75	2	Not recommended
6-12 wk	**62**	39	33	29	34	5	Undecided
12 wk to 6 mo	**86**	15	63	23	9	6	Recommended
>6 mo	**89**	12	78	11	5	7	Recommended

a Data from Thaler et al.^88^ Values are expressed as percentage of experienced shoulder surgeons surveyed.

b Sum of “allowed” and “allowed with experience.”

c Sum of “not allowed” and “no opinion” (based on the assumption of a more restrictive, safe view). Bold highlights represent the highest percentage of surgeons recommending or not recommending tennis in each time period.

Surgeons were more restrictive after TKA than after THA (Table 3). However, many studies allowed doubles play after THA and TKA, at least if the patient had experience, whereas singles tennis was sometimes not recommended or the authors were undecided.^70,86^

**Table 3:** Surgical Recommendation to Play Tennis After Total Knee Replacement*^*a*^*

Activity Postoperative Time Point	Yes*^*b*^*	No*^*c*^*	Allowed	Allowed With Experience	Not Allowed	No Opinion	Recommendation
Singles							
6 wk	11	**90**	2	9	82	8	Not recommended
6-12 wk	26	**74**	9	17	70	4	No opinion
12 wk to 6 mo	**62**	39	27	35	32	7	No opinion
>6 mo	**70**	31	41	29	23	8	No opinion
Doubles							
6 wk	16	**84**	4	12	77	7	Not recommended
6-12 wk	45	**55**	18	27	52	3	Not recommended
12 wk to 6 mo	**78**	23	45	33	18	5	Recommended
>6 mo	**87**	14	63	24	8	6	Recommended

a Data from Thaler et al.^87^ Values are expressed as percentage of experienced shoulder surgeons surveyed.

b Sum of “allowed” and “allowed with experience.”

c Sum of “not allowed” and “no opinion” (based on the assumption of a more restrictive, safe view). Bold highlights represent the highest percentage of surgeons recommending or not recommending tennis in each time period.

Some surgeons adapted their TKA implant choice if the patient wanted to play tennis again, favoring cruciate retaining implants with a highly congruent polyethylene design.^35^

The recommendations for TAA implantation are vague. Valderrabano et al^90^ and Macaulay et al^54^ did not allow singles and only allowed doubles with sufficient experience and equipment. Other authors considered tennis possible after TAA but did not differentiate between singles and doubles.^89^

Interestingly, of the surgeons surveyed in the questionnaire analysis by Macaulay et al,^54^ 56% were even more restrictive regarding any kind of sport for younger people (<50 years), 63% for patients with a body mass index >30, and 75% for those with poor bone quality. Other authors did not recommend tennis in cases of overweight.^35^ In contrast, 46% of surgeons were less restrictive for older people (>70 years). Experience played a major role, with around 40% of respondents allowing singles tennis and around 85% of those allowing doubles tennis for players with sufficient experience, whereas only 12% and 42%, respectively, allowed everyone to play singles and doubles tennis.^54^ Regardless of the medical requirements, however, significantly fewer people played tennis after TAA than before the operation.^2,66^

In general, TAA had a worse prognosis for RTP compared with arthroplasty in other joints. Similarly, no tennis player (0%) was able to return to the court after an ankle arthrodesis.^81^ Ankle arthrodesis is a complete salvage procedure and has a very low RTP rate due to functional limitations.

Regarding the shoulder joint, both singles and doubles were more likely to be allowed after SHA (74%-100%) or a-TSA (72%-97%), but surgeons were skeptical about sport after r-TSA (52%-75%).^55^ In another study, 84% of the surgeons surveyed allowed doubles tennis and 81% allowed singles tennis regardless of the type of prosthesis.^33^ Generally, doubles play was more likely to be allowed than singles play, which is particularly evident in r-TSA.^55^

There is some uncertainty as to whether doubles tennis is advisable after r-TSA in the study from Magnussen et al.^55^, whereas singles tennis should not be allowed. North American surgeons were more likely to allow tennis play than European surgeons, who were more restrictive^55^ (Table 4). Similar results from other authors showed that doubles and singles tennis play was possible after a-TSA.^17,39^

**Table 4:** Recommendation for Playing Tennis Depending on the Type of Shoulder Procedure*^*a*^*

			Allowed			Allowed With Experience			Not Allowed			Undecided		
Procedure and Activity	Yes*^*b*^*	No*^*c*^*	North America	Europe	All	North America	Europe	All	North America	Europe	All	North America	Europe	All
SHA														
Singles	**85**	15	60	33	46	36	41	39	4	25	15	0	0	0
Doubles	**88**	11	76	37	55	24	41	33	0	20	10	0	2	1
a-TSA														
Singles	**75**	25	40	29	33	38	43	42	19	29	24	2	0	1
Doubles	**86**	14	64	33	48	33	43	38	2	24	14	0	0	0
r-TSA														
Singles	30	**71**	11	10	10	11	26	20	75	60	67	3	5	4
Doubles	43	**58**	16	14	15	27	26	28	54	52	53	3	7	5

a Data from Magnussen et al.^55^ Values are expressed as percentage of experienced shoulder surgeons surveyed. Boldface indicates the most frequent citing. a-TSA, anatomic total shoulder arthroplasty; r-TSA, reverse total shoulder arthroplasty; SHA, shoulder hemiarthroplasty.

b Sum of “allowed” and “allowed with experience.”

c Sum of “not allowed” and “undecided” (due to the assumption of a more restrictive, safe view).

A return to singles tennis also appeared possible in individual cases after SHA due to rotator cuff arthropathy.^31^ Athletes with rheumatoid arthritis tended to have a lower RTP rate for any sport after SHA.^31^ A sport that requires external shoulder rotation and abduction, as is the case with tennis, can prevent RTP after r-TSA, even if players are motivated to play tennis again.^28^

Table 5 shows the percentage of tennis play clearance given by the surgeons surveyed depending on the type of prosthesis.

**Table 5:** Percentage of Tennis Play Clearance Given by Surveyed Surgeons Depending on the Type of Procedure*^*a*^*

Procedure and Activity	Tennis Clearance, %*^*b*^*
Total hip arthroplasty	
Single	80
Double	89
Total knee arthroplasty	
Single	70
Double	87
Total ankle arthroplasty	
Single	40
Double	85
Shoulder hemiarthroplasty	
Single	85
Double	88
Anatomic total shoulder arthroplasty	
Single	75
Double	86
Reverse total shoulder arthroplasty	
Single	30
Double	43

a Data from Macaulay et al,^54^ Magnussen et al,^55^ and Thaler et al.^87,88^

b Sum of “allowed” plus “allowed with experience.”

Another interesting question concerns the postoperative time to resume playing tennis. Although a few surgeons allowed RTP at just 6 weeks postoperatively after THA or TKA, the percentage increased significantly after the sixth postoperative month (Tables 2 and 3^87,88^). However, the data are limited. The recommendations appear to be given for singles play at 6 months after THA and for doubles play after 3 months.^88^ We found no consensus for singles play after TKA but noted a recommendation for doubles play after 3 postoperative months.^87^

In a follow-up questionnaire after THA, 58 active tennis players reported that they had played competitive tennis at a mean of 6.7 months (range, 1-12 months) postoperatively. A similar survey after TKA in 33 patients showed a return to competitive tennis after a mean of 5.9 months (range, 1-10 months).^61,63^ It is interesting to note that 21% and 30% of those surveyed had undergone THA or TKA, respectively, primarily to be able to play tennis again.^61–63^ Similarly, more people played tennis after THA and TKA than preoperatively^40^ (possibly due to preoperative pain).

Therefore, there appears to be a certain bias in both studies from Mont et al., since it seems that the questionnaire was filled out by people who were particularly fond of tennis.^61,63^ For example, an elite Association of Tennis Professionals (ATP) player aged 40 was able to play tennis 5.5 months after a hip resurfacing operation.^64^ Yet, a hip resurfacing procedure is a surgery for younger and more active patients, in contrast to the standard THA patient. Many patients can return to a variety of sports after hip resurfacing procedures, even professional tennis, as widely known from elite player Andy Murray.

Four tennis players who had undergone hip replacement returned to the tennis court after a significantly longer period (almost 20 months; range, 17-23 months).^14^ However, experience shows that despite the possibility to play early after surgery and the clearance to play, it often takes 9 to 12 months until coordination and muscular rehabilitation allows responsible RTP, especially in TKA but also THA.^45^ For example, it was shown that quadriceps strength needed at least 6 months to recover after TKA.^74^

Although data were pooled with different sports, Magnussen et al^55^ showed that most of the surgeons surveyed (50%-60%) allowed RTP after about 5 to 7 months, regardless of the type of shoulder prosthesis. Less than 10% considered this possible only after 8-10 or 11-12 months, whereas about 40% considered this possible at 2 to 4 months after SHA and about 20% after a-TSA or r-TSA.^55^

Pooled data for different sports including tennis showed RTP on average after 5.5 to 6.5 months (range, 1-24 months) for SHA,^29,31,52^ after 5.4 to 6.7 months (range, 1-12 months) for a-TSA,^29,30^ and after 3.4 to 5.3 months (range, 1-36 months) for r-TSA.^21,32,52^ Partial tennis practice was possible on average after 3.6 months (pooled data for SHA and a-TSA) and full practice was possible only after 5.8 months.^59^

One study found that after r-TSA, 70% of participants could start playing tennis after just 3 to 6 months postoperatively, with 30% needing 9 to 12 months to get back on the court. Further, 70% of players played tennis without any restrictions after just 6 to 9 months, whereas 30% need >2 years to do so.^48^

Table 6 shows the time required to return to tennis depending on the type of procedure and the surgeon's approval.

**Table 6:** Period to Return to Play After Procedure and Surgeon Approval*^*a*^*

Procedure	Period to RTP	Permission According to Surgeon
THA	6.7 mo (1-12 mo)	Singles 6 mo, doubles 3 mo
TKA	5.0-6.9 mo (1-12 mo)	Singles no consensus, doubles 3 mo
UKA	2.5 mo (2-3 mo)	NA
TAA	NA	NA
SHA	5.5-6.5 mo (1-24 mo)	2-4 mo, 5-7 mo*^*b*^*
a-TSA	5.4-6.7 mo (1-12 mo)	2-4 mo, 5-7 mo, 8-10 mo*^*c*^*
r-TSA	3.4-5.3 mo (1-36 mo)	2-4 mo, 5-7 mo, 11-12 mo*^*d*^*

a Data from references 11, 15, 21, 29-32, 52, 55, 61, 62, 87, 88, and 95. a-TSA, anatomic total shoulder arthroplasty; NA, not applicable; r-TSA, reverse total shoulder arthroplasty; RTP, return to play; SHA, shoulder hemiarthroplasty; TAA, total ankle arthroplasty; THA, total hip arthroplasty; TKA, total knee arthroplasty; UKA, unicondylar knee arthroplasty.

b 40% allowed tennis after 2-4 mo, 50%-60% after 5-7 mo.

c 20% allowed tennis after 2-4 mo, 50%-60% after 5-7 mo, 10% after 8-10 mo.

d 20% allowed tennis after 2-4 mo, 50%-60% after 5-7 mo, 10% after 11-12 mo.

### Ability to Play

The question remains as to how a joint prosthesis affects tennis playing ability. At the time of examination after prosthesis implantation (THA 8 years [range, 2-22 years]; TKA 7 years [range, 2-18 years]), most players in an evaluation played tennis on average 3 times a week (range 1-7 times per week).^61,62^ A different study on TKA reported that 4 players (15%) played <3 hours per week, 10 players (37%) 4 to 6 hours per week, 4 players (15%) 7 to 9 hours per week, and 9 players (33%) 10 to 15 hours per week. The exertion level was very light for 1 player (4%), light for 7 players (26%), moderate for 14 players (52%), and vigorous for 5 players (19%).^51^

Mont et al^61,62^ reported an improvement in mobility parameters and playing ability in tennis for both THA^61^ and TKA^62^ (Table 7). Although the patients examined had certain difficulties in various tennis-specific game variables preoperatively, including joint stiffness and/or pain, these symptoms improved significantly postoperatively. Among other things, strokes could be swung through better after THA and TKA, weight shifting was better coordinated, and movement on the court improved (Table 4). At the same time, however, running speed on the court decreased after both THA and TKA. After THA or TKA, players took anti-inflammatory medication before playing in 26% (THA) and 21% (TKA) of cases. However, significantly more players (90% and 91%) had to take pain-reducing medication preoperatively. Patients after THA in particular had load-related thigh pain, which could be positively influenced by medication.^61,62^ A different study stated that 33% of players could only play for a shorter time period, whereas 39% could play for the same time and 28% could play for a longer period after TKA. The tennis swing of these players did not change in 69%, improved in 17%, and worsened in 14%. Two-thirds (66%) played only doubles, and 17% each played either only singles or both.^51^

**Table 7:** Improvement of Tennis Playing Parameters After Surgery*^*a*^*

Parameter	Preoperative, THA/TKA	Postoperative, THA/TKA
Bending to hit a shot at waist level	2.9/2.8	4.2/4.2
Follow-through		
Forehand	2.8/2.9	4.3/4.1
Backhand	2.8/2.8	4.3/4.1
Volley	2.8/2.9	4.3/4.2
First serve	2.8/2.7	4.3/4.2
Second serve	2.8/2.7	4.3/4.2
Shifting weight		
Forehand	2.4/2.2	4.4/4.2
Backhand	2.4/2.1	4.4/4.1
Volley	2.4/2.3	4.4/4.4
First serve	2.8/2.6	4.2/4.3
Second serve	1.5/2.4	4.4/4.4
Overheads	1.3/1.6	4.4/4.1
Move side to side on the baseline	2.0/1.8	4.2/4.1
Move forward after serve to volley	2.0/2.3	3.2/3.4
Stop abruptly and turn direction	2.4/2.2	4.0/3.8
Hit a ground stroke on the run	1.0/1.4	3.8/3.5
Play one 3-set match of singles	2.1/1.7	4.0/3.5
Play one 3-set match of doubles	2.1/2.3	4.0/4.2
Play on hardcourt	2.5/2.1	4.0/4.1
Play on grass	2.5/2.6	3.0/3.2
Play on clay	2.5/2.8	4.0/4.4

a Values are based on the following scale: 1 = cannot perform; 2 = extremely difficult to perform (pain and stiffness); 3 = difficult to perform (stiffness only and slight pain); 4 = can perform with some stiffness and no pain; 5 = can perform with no stiffness and no pain. Data from Mont et al.^61,63^ THA (total hip arthroplasty), n = 58 patients/75 arthroplasties; TKA (total knee arthroplasty), n = 33 patients/46 arthroplasties.

An elite-level ATP player was able to play 53 doubles tennis matches on the ATP Tour at the age of 40 after a hip resurfacing operation. Although he won 70% of his matches, his performance showed a downward trend, so that he withdrew from the tour shortly afterward.^64^

In another study, the inability to play doubles tennis postoperatively was due, among other things, to a possible hip abductor contracture or weakness as well as an obvious leg-length discrepancy after a hip resurfacing operation. After a knee replacement, the impairment was due to a possible knee flexion contracture, a knee flexion deficit of <90°, and quadriceps muscle weakness.^6^

Considering the low RTP rate after an ankle replacement, the ability to play appears to be significantly reduced. In a questionnaire study, 82% of patients stated that they could no longer play tennis due to ankle problems. Only 7 patients (18%) could play tennis, and they did this only occasionally.^10^ Overall, the number of patients who had ankle replacement and played tennis was very low.^10,90^

The data seem insufficient to make clear, evidence-based statements about the ability to play tennis after TAA. The majority of patients had corresponding ankle problems or pain when exercising that prevented them from playing tennis (especially competitively) after surgery.^10^

Competitive tennis no longer seemed possible after a-TSA of the dominant arm due to limited joint mobility and reduced strength. However, postoperative recreational tennis with a mean playing time of 80 minutes 2 times per week (range, 1-3 times) was manageable, despite a limited subjective range of motion of 88% (75%-100%) compared with before shoulder disease (subjective range of motion 100% before the shoulder disease/declined during the shoulder disease to 57% (50%-70%)). However, the nondominant side was pooled in the data with the hitting arm.^82^

In another pooled group of 20 tennis players, racquetball players, and overhead throwers after a-TSA, only 50% reported that their level of play was the same as before the operation, whereas 29% had a poorer level of play and 21% were unable to practice their sport.^57^ This study emphasized an important point in outcomes research in sports medicine: the reporting of return to sport versus return to sport at equal or higher level than before the operation and/or injury. These metrics can be vastly different and must be specifically clarified to optimize data interpretation from these types of studies. A different questionnaire study reported that tennis was among the main activities patients wished to perform but were unable to after a-TSA.^96^

After a joint replacement, it may be advisable to undertake training to adapt one's tennis technique or hitting technique accordingly. The aim is to reduce the twisting and bending load on the new hip or knee joint with each stroke, so that each stroke (serve, forehand, backhand, and volley) is carried out in a way that is gentle on the joints. The serve should be performed standing upright and without jumping forward to reduce the load on the prosthesis. With both the forehand and backhand strokes, the player should not slide into the ball but should move toward the ball in small steps. The swings should be kept shorter, from a more open position, without excessive forward thrust of the legs and with less bending of the knee, so that the twisting and shearing load on the new hip and knee joint is reduced.^45^

Furthermore, patients should consider refraining from serving after a shoulder endoprosthesis implantation and playing tennis only recreationally without competitive ambition.

### Complications

The reason why some surgeons prohibit singles tennis in particular is the fear of prosthesis-associated complications, such as aseptic loosening, early polyethylene wear, or a periprosthetic fracture with early subsequent revision surgery. Movements including joint impingement, distortion, subluxation, or microseparation through a high range of motion or fast impulse and acceleration may result in early polyethylene wear.

Higher rates of failure as well as loosening and polyethylene wear were observed in high-impact sports of the lower extremity after THA^16,37,56^ and TAA.^4,36,83^ No data on higher failure rates in TKA could be found in the literature, and Kornuijt et al^49^ could not find any difference between patients with a high and low physical activity level. Although some studies have shown higher revision and wear rates after arthroplasty in highly active individuals,^20,25,27,34,47,56^ other studies found no negative influence of high-impact sports or tennis on implant longevity in THA,^¶^ TKA,^19,49^ and TAA.^44,66^ No increased ion levels could be measured in a professional ATP player after a hip resurfacing operation.^64^

In a selected population of tennis players, the revision rate after both THA and TKA was 4% (n = 3 and n = 2, respectively).^61,62^ In the case of TKA, the revisions were carried out because of polyethylene wear 8 and 11 years after the primary implantation, respectively.^62^ The affected patients were clinically asymptomatic at the follow-up 5 and 7 years after the revision surgery and were able to play tennis up to 5 times a week.^62^

Even after TAA, no relevant negative effects were seen from performing sporting activities.^10^ However, because the risk of aseptic loosening after TAA may be higher in high-impact sports, patients should at least wait for the bone healing phase to complete (9 months) before starting to play sports again.^86^

Acromion fractures, rotator cuff lesions, joint dislocations, component loosening, periprosthetic fractures, and wear were frequently described complications after shoulder prosthesis implantation.^8,9,53,85^ However, in a pooled collective of almost 100 r-TSA and 70 SHA procedures, no sport-associated complications could be seen in the midterm,^52^ similar to many other studies after a-TSA, r-TSA, and SHA.^30-32,53,85^ One study found a radiologically visible loosening margin in 1 zone (using the Gruen classification) in 17% of cases but without shaft loosening after r-TSA. Furthermore, no glenoid loosening margins or loosening were seen in the same collective.^85^ Interestingly, the risk of glenoid wear appeared to be higher after SHA than after a-TSA. The outcome after conversion of SHA to a-TSA seemed to be worse in 1 study, so Barlow and Abboud^5^ recommended a-TSA instead of SHA for young, active patients. During the literature review, no study could be found that linked tennis to a higher complication rate after implantation of a shoulder prosthesis.

In contrast to the possible negative consequences of playing tennis after having arthroplasty, the positive effects of participating in tennis should also be mentioned. These include general protective effects of sport such as weight reduction, better adaptation to fears, higher cerebral endorphin release, improvement of back pain and cardiovascular risks, and better control of hypertension, diabetes mellitus, and osteoporosis.^84^

Tennis has significant health benefits. Oja et al^69^ reported that it can contribute to a 47% reduction in the risk of overall mortality and a 59% reduction in the risk of mortality from a heart attack or stroke. Yet, this is probably due to the general lifestyle as well as the social higher social status of this cohort and the overall benefits of exercise.

Tennis players showed an increase in life expectancy of 9.7 years compared with sedentary people and led in terms of life extension ahead of cyclists (increase of 3.7 years), swimmers (increase of 3.4 years), joggers (increase of 3.2 years), and health club participants (1.5 years).^80^

### Study Assessment and Limitations

The existing evidence base is limited with regard to the research question. The follow-up period is often very short and the level of evidence is mostly low. Basically, the literature consists of expert opinions, retrospective studies, and questionnaire studies without control groups or randomization. The level of evidence (LoE) is 3 to 5 for all procedures (THA: LoE 3 [n = 4], LoE 4 [n = 3], LoE 5 [n = 1]; TKA: LoE 3 [n = 6], LoE 4 [n = 3]; UKA: LoE 3 [n = 3], LoE 4 [n = 2]; TAA: LoE 3 [n = 1], LoE 4 [n = 2]; a-TSA: LoE 3 [n = 1], LoE 4 [n = 3]; r-TSA: LoE 3 [n = 3], LoE 4 [n = 2]; SHA: LoE 3 [n = 2], LoE 4 [n = 1]; SA: LoE 3 [n = 3], LoE 4 [n = 1]). Systematic reviews and meta-analyses included studies of LoE 3 and 4 and were graded LoE 3 (Appendix Table A1). Often the number of cases was small, or patients and their sport practice were pooled in the literature—as is the case with the terms *low-impact* and *high-impact* sport, for example. The same applied to the partial lack of differentiation between singles (which is classified as a high-impact sport) and doubles (which is classified as a low-impact sport). Furthermore, a certain selection bias was present; for example, in the tennis-specific studies by Mont et al,^61,63^ primarily highly motivated players were surveyed, some of whom played 5 times a week after arthroplasty. It is doubtful whether such results can be extrapolated to the general population or to all patients after THA or TKA who want to play tennis. In the short-term follow-up periods, no harmful effects of tennis could be proven, but long-term studies are lacking to accurately assess the effect of tennis on THA and TKA.^84^

We believe that each patient should be assessed and advised individually and also informed that the practice of high-impact sports (such as tennis) could potentially lead to complications like premature polyethylene wear, implant loosening, or periprosthetic fractures, even though no fully conclusive data are available at this time.

After any shoulder endoprosthesis implantation, the follow-up period of the studies appeared to be too short to assess complications such as the risk of prosthesis loosening, rotator cuff lesions, or periprosthetic fractures due to sporting activity or tennis playing.^57,72^ Furthermore, reports on shoulder prosthesis implantations rarely differentiated between the hitting arm side and the contralateral side.

Finally, there is a lack of multicenter prospective and randomized data that examine homogeneous male and female tennis players to make high-quality evidence-based statements or recommendations. Regarding THA, a systematic review of the outcomes, including RTP, is planned and was registered with the National Institute of Health Research Prospective Register of Systematic Reviews in 2025.^46^

## Conclusion

In view of the current evidence, tennis may be continued even after arthroplasty. The RTP rate seemed higher for total hip arthroplasty, total knee arthroplasty, anatomic shoulder arthroplasty, and shoulder hemiarthroplasty compared with ankle and reverse shoulder arthroplasty. The time frame for a return to sports depends on a variety of intrinsic and extrinsic factors. For players with sufficient experience, tennis doubles and singles may be played after 3 to 6 months. Each patient should be assessed and advised individually and also informed that the practice of high-impact sports (such as tennis) could potentially lead to complications like premature polyethylene wear, implant loosening, dislocations, or periprosthetic fractures, even though no fully conclusive data are available. This report can provide physicians with information about the outcomes and RTP rates for tennis after shoulder, hip, knee, and ankle replacement. It will help physicians provide better advice to patients who wish to return to tennis after endoprosthesis surgery

## Appendix

**Appendix Table A1:** Level of Evidence of Included Studies Regarding Return to Tennis Play After Arthroplasty*^*a*^*

Procedure and Level of Evidence	References
Total hip arthroplasty	
LoE 3	Chatterji et al^14^ Huch et al^40^ Innmann et al^42^ Pasqualini et al^75^
LoE 4	Dubs et al^26^ Schmidutz et al^79^ Wylde et al^97^
LoE 5	Oljaca et al^70^
Total knee arthroplasty	
LoE 3	Chatterji et al^15^ Huch et al^40^ Pasqualini et al^76^ Vielgut et al^92^ Walton et al^95^ Lawrence et al^51^
LoE 4	Bock et al^7^ Bradbury et al^11^ Wylde et al^97^
Unicondylar knee arthroplasty	
LoE 3	Papalia et al^73^ Pietschmann et al^77^ Walton et al^95^
LoE 4	Naal et al^65^ Walker et al^94^
Total ankle arthroplasty	
LoE 3	Schuh et al^81^
LoE 4	Bonnin et al^10^ Naal et al^66^
Anatomic total shoulder arthroplasty	
LoE 3	Garcia et al^29^
LoE 4	Garcia et al^30^ Neer et al^67^ Schumann et al^82^
Reverse total shoulder arthroplasty	
LoE 3	Davey et al^21^ Kolling et al^48^ Liu et al^52^
LoE 4	Fink Barnes et al^28^ Garcia et al^32^
Shoulder hemiarthroplasty	
LoE 3	Garcia et al^29^ Liu et al^52^
LoE 4	Garcia et al^31^
Shoulder arthroplasty	
LoE 3	Aim et al^1^ Liu et al^53^ Papalia et al^72^
LoE 4	McCarty et al^59^

a Systematic reviews and meta-analyses included studies with level of evidence (LoE) 3 and 4 and were graded 3.

## Notes

¶ References 11, 17, 18, 23, 26, 50, 60, 61, 64, 68, 78, 93.
